# Prognostic Value of microRNA-9 in Various Cancers: a Meta-analysis

**DOI:** 10.1007/s12253-016-0148-4

**Published:** 2016-11-14

**Authors:** Yunyuan Zhang, Jun Zhou, Meiling Sun, Guirong Sun, Yongxian Cao, Haiping Zhang, Runhua Tian, Lan Zhou, Liang Duan, Xian Chen, Limin Lun

**Affiliations:** 1 Department of Clinical Laboratory, The Affiliated Hospital of Qingdao University, Qingdao, 266003 People’s Republic of China; 2 College of Laboratory Medicine, Key Laboratory of Laboratory Medical Diagnostics Designated by Chinese Ministry of Education, Chongqing Medical University, Chongqing, 400016 People’s Republic of China; 3 Department of Laboratory Medicine, the Second Hospital Affiliated to Chongqing Medical University, Chongqing, 400010 People’s Republic of China

**Keywords:** miR-9, Cancer, Prognosis, Meta-analysis

## Abstract

Recently, there are more and more evidences from studies have revealed the association between microRNA-9 (miR-9) expression and outcome in multiple cancers, but inconsistent results have also been reported. It is necessary to rationalize a meta analysis of all available data to clarify the prognostic role of miR-9. Eligible studies were selected through multiple search strategies and the quality was assessed by MOOSE. Data was extracted from studies according to the key statistics index. All analyses were performed using STATA software. Twenty studies were selected in the meta-analysis to evaluate the prognostic role of miR-9 in multiple tumors. MiR-9 expression level was an independent prognostic biomarker for OS in tumor patients using multivariate and univariate analyses. High expression levels of miR-9 was demonstrated to associated with poor overall survival (OS) (HR = 2.23, 95 % CI: 1.56–3.17, *P* < 0.05) and recurrence free survival/progress free survival (RFS/PFS) (HR = 2.08, 95 % CI: 1.33–3.27, *P* < 0.05). Subgroup analysis showed that residence region (China and Japan), sample size, cancer type (solid or leukemia), follow-up months and analysis method (qPCR) did not alter the predictive value of miR-9 on OS in various cancers. Furthermore, no significant associations were detected for miR-9 expression and lymph node metastasis or distant metastasis. The present results suggest that promoted miR-9 expression is associated with poor OS in patients with general cancers.

**Electronic supplementary material:**

The online version of this article (doi:10.1007/s12253-016-0148-4) contains supplementary material, which is available to authorized users.

## Introduction

Cancer is not only the most serious disease but also the leading cause of death for human health over the past decade [1]. In 2014, an estimated 1,665,540 new cancer cases and 585,720 cancer deaths are projected to occur in the United States [2]. Even though tremendous progresses have been made in recent years, tumor node metastasis (TNM) stage and grade, tumor size, demographics, high rates of recurrence and drug resistance are still widely used to determine the prognosis of cancer. Currently, the mechanism of oncogenesis and tumor progression is still not fully elucidated, which restrict the prognosis and metastasis prediction of cancer patients. Many scientists endeavored to cancer diagnosis, prognosis, and treatment because effective prognostic markers and therapeutic methods used for tumor therapy and prevention have not been discovered. Some researchers had suggested that identifying ideal prognostic markers in cancer would be valuable for proper individual management. Therefore, it is critical to finding a convenient and effective biomarker with high accuracy prognostic value for cancer patients to improve the survival status.

As a class of endogenous, evolutionarily conserved small non-coding RNAs, microRNAs (miRs) have a crucial role that destabilize the target protein-coding mRNAs or inhibit their translation by interacting with complementary sequences [3]. Since the discovery of miRs in 1993, growing evidence reveals that miRs not only regulate multiple biological processes, but also associated with tumor-genetic procedures, such as adhesion, migration, invasion, angiogenesis and apoptosis [4]. Over the last decade, miRs have gained great attention as novel biomarkers in tumor prognosis because lots of miRs are aberrantly expressed in multitudes of cancers [5]. Hence, more and more researches focus on the miRs as the promising biomarkers of prognosis.

In recent years, a host of miRs biomarkers have been investigated in cancer. Among them, miR-9 has been considered as a candidate prognostic factor in different cancer. Many observations indicate that the promoted miR-9 level was associated with worse survival and high risk of cancer metastasis in patients with various carcinomas. However, consensus has not been reached as to the credibility of miR-9 as a prognostic biomarker in tumor because some other studies presented insignificant or inverse results. Thus, a comprehensive meta analysis of all eligible literatures was conducted to further assess the clinical feasibility of miR-9 as a novel biomarker for tumor prognosis.

## Material and Methods

The meta analysis was performed totally following the guide lines of Preferred Reporting Items for Meta analysis of Observational Studies in Epidemiology group (PRISMA). Review protocol could be accessed on the site http://www.crd.york.ac.uk/PROSPERO/ with registration number CRD42016032714.

### Search Strategy and Literature Selection

The Cochrane Library, PubMed (medline), Embase, ISI Web of Knowledge, ScienceDirect, BioMed Central, Springer together with three Chinese databases: Weipu, Wanfang and China National Knowledge Internet (CNKI) databases were used to conduct a comprehensive computerized literature search for articles that evaluated the accuracy of miR-9 for the prognosis and metastasis of cancer. The studies were identified by using the following keywords in variably combinations: (“microRNA-, miRNA-, miR-9,”) and (“cancer” or “tumor” or “tumour” or “neoplasm”) and (“prognostic” or “prognosis” or “survival” or “recurrence” or “metastasis”). In addition to the electronic literatures that published between inception and July 1, 2016, the reference lists of primary studies were also searched for additional articles.

### Inclusion and Exclusion Criteria

Inclusion criteria: 1) Studied the patients with any malignant tumor; 2) Definite diagnosis confirmed for patients with cancer; 3) Studies appraising miR-9 in tissues or serum for cancer; 4) Studies with sufficient information to construct the 2 × 2 contingency Table.

Exclusion criteria: 1) Literatures not pertinent to the miR-9; 2) Studies of non dichotomous miR-9 expression and absence of survival outcome; 3) Similar studies from the same author as well as multiple duplicate data in the different works, excluding earlier and smaller sample data; 4) Animal experiments, case reports, correspondences, reviews, expert opinions, letters, talks, and editorials without original data.

### Quality Assessment

As shown in supplementary materials (Checklist S1), all the eligible articles were systematically assessed based on a critical review checklist of the Dutch Cochrane Centre proposed by MOOSE [6]. Major items to be evaluated are as following: (I) enough information of all types of cancer, (II) clear description of study design, (III) well defined cancer outcomes (IV) clear description of miR-9 measurement, and (V) sufficient period of follow-up. Articles should be excluded if the necessary information mentioned above can not be obtained.

### Data Extraction

Data was carefully extracted from all eligible studies in duplicate by two independent investigators (YYZ and XC). Extracted databases were crosschecked between the two authors to rule out any discrepancy. Disagreement was dissolved by consulting with a third investigator (LML). The following data for each collected studies were extracted independently: (I) first author, publication year, study population, and the patients number; (II) miR-9 assay specimen, miR-9 assessment method; (III) HR and their 95 % CI of miR-9 value for overall survival, lymph node metastasis or distant metastasis, with an HR of >1 being associated with a poorer outcome. If only Kaplan–Meier curves were presented in some articles, the statistical variables were extracted from the graphical survival plots and an estimation of HR value was then calculated as the previously described methods [7, 8]. If any essential information were not available from the article, best efforts were made to sending a reminder to the corresponding authors.

### Statistical Analysis

All analyses were performed using STATA software, version 12 (Stata Corporation, College Station, TX, USA). The intensity of relationship between the miR-9 expression and outcome were described as Hazard ratio (HR). All HR and 95 % confidence interval (CI) were combined from the eligible articles, and RR was directly considered as HR. Heterogeneity was quantified with the *I* ^*2*^ metric. Heterogeneity across the eligible studies was also tested using the Q-test, and the results were considered statistically significant when *P* value less than 0.05. A random effects model was used to evaluate the relationship between miR-9 expression and survival or metastasis when there was significant heterogeneity between the included studies (*I* ^*2*^ > 30 %). Since publication bias is critical to the meta analyses, potential publication bias was assessed by a funnel plot, Begg’s and Egger’s bias indicator test. Sensitivity analysis was performed to evaluate the stability of the results.

## Results

### Screening of the Literature

**Fig. 1:**
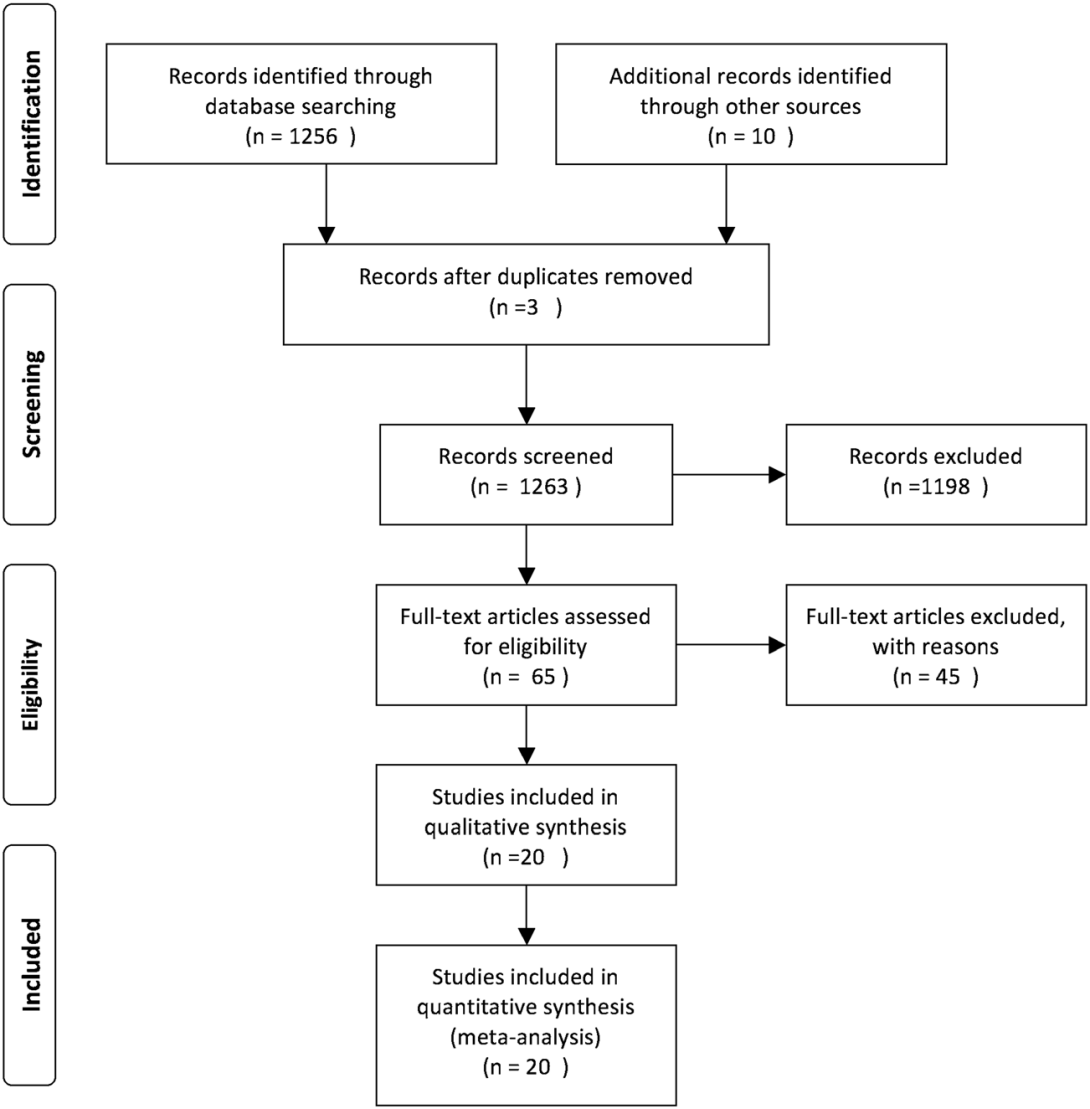
Flow chart of the study selection process

A total of 1266 studies were retrieved from an initial online literature search that related to the prognosis and metastasis of miR-9 and cancer. Based on screening titles and abstracts of focused articles, 1198 articles were excluded according to the inclusion and exclusion criteria. The full text of the remaining 65 articles were further identified, 45 studies were excluded because miR-9 was not treated as a dichotomic variable. Finally, the remaining 20 publications [9–28] were combined in the meta analysis and the selection process presented by a flow chart which is shown in Fig. 1.

### Characteristics of the Analyzed Studies

The main characteristics of the analyzed studies were summarized in Table 1. In summary, all the 20 studies were retrospective, which dealt with a total of 2441 patients from the United States, German, France, China, Japan and Korea et al. The patients were of 14 kinds of carcinomas, including esophageal cancer, ovarian carcinoma, bladder tumors, glioma, non-small cell lung cancer, human laryngeal squamous cell carcinoma, osteosarcoma, adrenocortical cancer, hepatocellular carcinoma, breast cancer, nasopharyngeal carcinoma, squamous cell carcinoma, leukemia and colorectal cancer. Different kinds of cancerous tissues were usually examined to determine miR-9 expression level, most by qRT-PCR methods, while serum samples were tested in one study. Of note, the median value was selected as the cut-off value in most articles.

**Table 1:** Summary table of the 20 included studies

Study	Origin of population	Study design	Disease	N	Stage	miR-9 assay	Survival analysis	Metastasis analysis	Hazard ratios	Follow-up months
Hu 2010	USA	R	EC	172	NA	MISH	OS/DFS	NA	HR	150
Maki 2012	Japan	R	Leukemia	124	NA	qRT-PCR	OS/RFS	NA	HR/K-M	17(median)
Li 2013	China	R	OC	45	I-IV	qRT-PCR	OS/DFS	NA	K-M	80
Pignot 2013	France	R	BLC	72	II-IV	qRT-PCR	OS/RFS	NA	K-M	72
Wu 2013	China	R	Glioma	128	I-IV	qRT-PCR	OS	DM	HR/ K-M	60
Xu 2013	China	R	NSCLC	116	Ia-Ib/IIa-IIb/IIIa	qRT-PCR	OS/PFS	NA	HR/ K-M	36
Sugita 2014	Japan	R	ALL	55	NA	qRT-PCR	OS/RFS	NA	HR/ K-M	60
Wu2014	China	R	LSCC	103	I-IV	qRT-PCR	OS	LNM	HR/ K-M	60
Xu 2014	China	R	OSA	79	I-II/III	qRT-PCR	OS	DM	HR/ K-M	60
Song 2014	China	R	EC	243	I-II/III-IV	MISH	OS	LNM	HR/ K-M	60
Fei 2014	China	R	OSA	118	I–IIA/IIB-III	qRT-PCR	OS	NA	K-M	80
Cai 2014	China	R	HCC	200	I-II/III-IV	qRT-PCR	OS	NA	HR/ K-M	60
M.Faria 2015	German	R	AC	28	I-IV	qRT-PCR	OS/DFS	DM	K-M	100
Zhang 2015	TCGA	R	HCC	327	I-IV	qRT-PCR	OS	NA	K-M	166
Gwak 2014	Korea	R	BC	166	I-II/III	qRT-PCR	DFS	NA	HR/ K-M	120
Sun 2015	China	R	HCC	60	I-II/III-IV	qRT-PCR	DFS	NA	HR	20
Lu 2013	China	R	NC	150	I-IV	qRT-PCR	NA	LNM/DM	NA	NA
White 2013	USA	R	ESCC	139	NA	MISH	NA	LNM	NA	NA
Feng 2014	China	R	EC	50	I-II/III-IV	qRT-PCR	NA	LNM	NA	NA
Long 2014	China	R	CRC	66	I-II/III-IV	qRT-PCR	NA	LNM/ DM	NA	NA

Study design is described as retrospective (R)

*BC*, breast cancer, *OSA* osteosarcoma, *NSCLC* non-small cell lung cancer, *CRC* colorectal cancer, *HCC* hepatocellular carcinoma, *BLC* bladder cancer, *OC* ovarian carcinoma, *EC* esophageal cancer, *LSCC* laryngeal squamous cell carcinoma, *AC* Adrenocortical cancer, *ESCC* epithelia squamous cell carcinomas, *NC* nasopharyngeal carcinoma, *MISH* microRNA in situ hybridization, *DM* distant Metastasis, *LNM* Lymph Node Metastasis, *TCGA* TCGA data portal

### miR-9 and Overall Survival

**Fig. 2:**
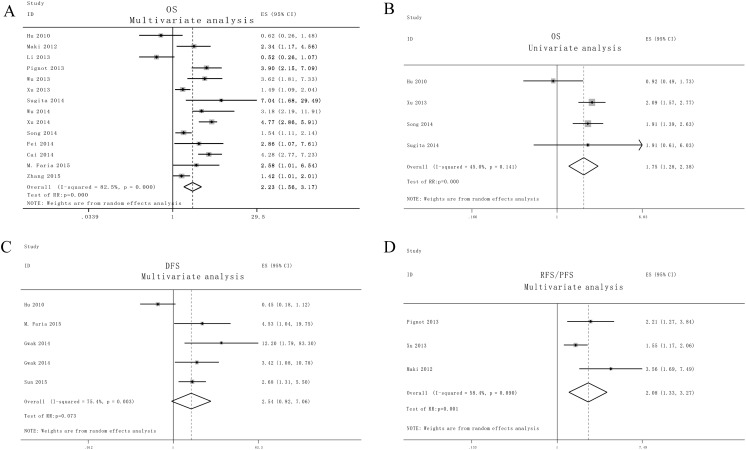
Forrest plots of studies evaluating hazard ratios of high miR-9 expression as compared to low expression. Survival data are reported as multivariate analysis of OS (**a**), univariate analysis of OS (**b**), disease-free survival (DFS) (**c**) and relapse-free survival (RFS) or progress-free survival (PFS) (**d**)

A random model was applied to calculate the pooled HR and its 95 % CI due to the presence of heterogeneity among the studies which involved in overall survival (OS) (*I* ^*2*^ > 30.0 %). High expression levels of miR-9 was demonstrated to significantly predict unfavorable OS in various human cancers, with the pooled HR of 2.23 (95 % CI: 1.56–3.17, *P* < 0.05) in multivariate analysis studies (Fig. 2a). Similarly, four univariate analysis studies also predict the association between high expression levels of miR-9 and OS (HR: 1.75, 95 % CI: 1.28–2.38, *P* < 0.05) (Fig. 2b).

**Fig. 3:**
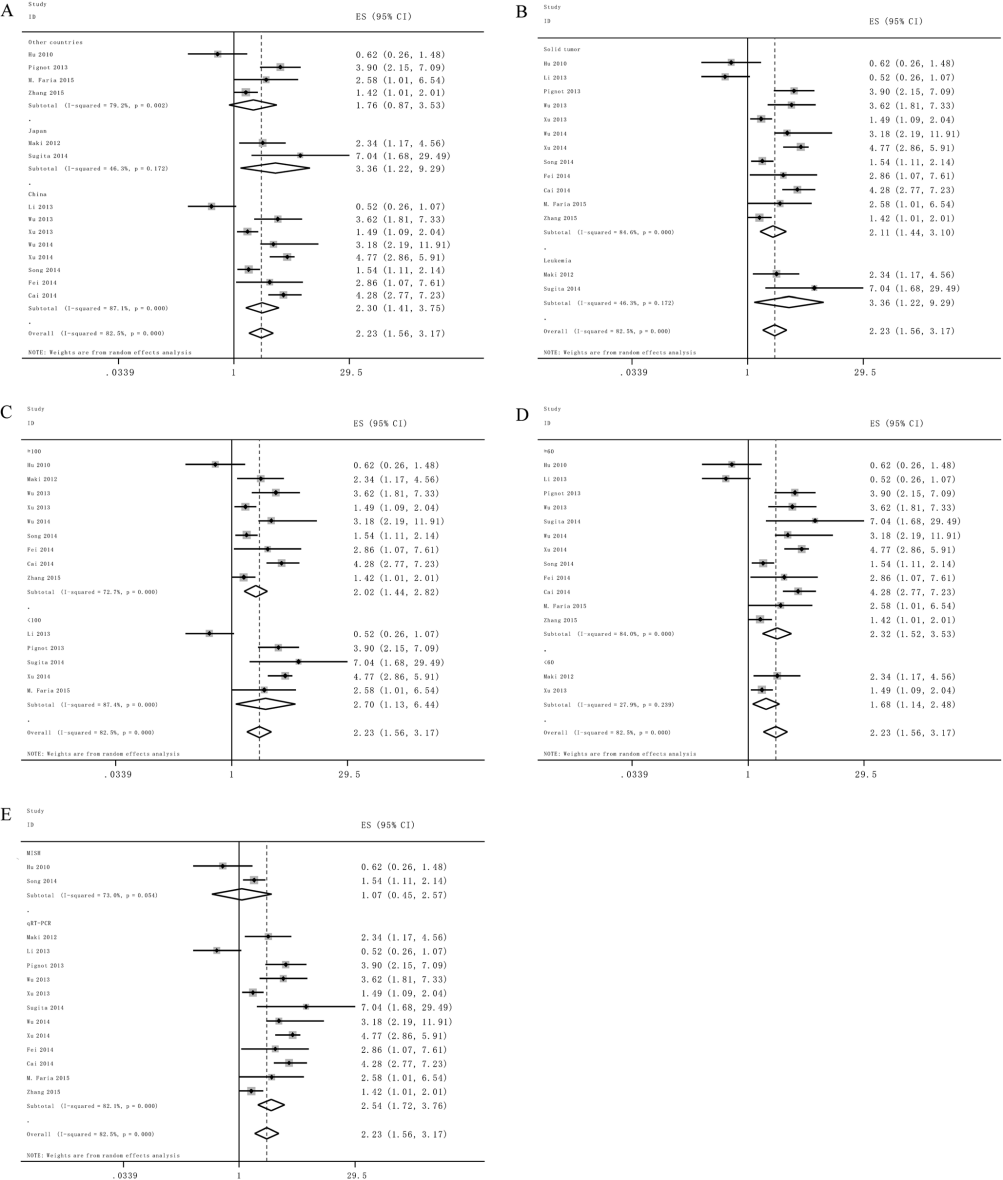
Forest plot showing the subgroup analyses of the pooled HRs with elevated miR-9 expression in the different types of cancer. Values of p and I^2^ and the HRs with their 95 % CI of overall survival (OS) were analyzed by the factors of Country (**a**), Cancer type (**b**), sample size (**c**), Follow-up month (**d**), Method (**e**)

Afterwards the subtotal analyses were performed by the countries, the cancer types, the sample sizes, and the analysis methods to analyze the possible sources of the heterogeneity (Table 2). The elevated miR-9 expression predicted a significantly worse OS with cancers in China (HR = 2.30; 95 % CI: 1.41–3.75, *P* < 0.05) and Japan (HR = 3.36; 95 % CI: 1.22–9.29, *P* < 0.05) (Fig. 3a). There was significant heterogeneity across the studies within the subgroups.

**Table 2:** Subgroup analysis of the pooled HRs of overall survival with overexpressed miR-9 in patients with cancer

Subgroup analysis	No. of studies	No. of patients	Pooled HR (95 % CI)		Heterogeneity (random)	
			Random *p* Value		I^2^(%) *p* Value	
Region						
China	8	1032	2.30(1.41,3.75)	0.001	87.1	0.000
Japan	2	179	3.36(1.22,9.29)	0.019	46.3 %	0.172
Other countries	4	599	1.76(0.87,3.53)	0.115	79.2 %	0.002
Sample size						
≥100	9	1531	2.02(1.44,2.82)	0.000	72.7 %	0.000
<100	5	279	2.70(1.13,6.44)	0.025	82.5 %	0.000
Type of cancer						
Solid cancer	12	1631	2.11(1.44,3.10)	0.000	84.6 %	0.000
Leukemia	2	179	3.36(1.22,9.29)	0.019	46.3 %	0.172
Type of methods						
MISH	2	415	1.07(0.45,2.57)	0.875	73 %	0.054
qRT-PCR	12	1621	2.23(1.56,3.17)	0.000	82.1 %	0.000
Follow-up years						
≥60	12	1570	2.32(1.51,3.53)	0.000	84 %	0.000
<60	2	240	2.23(1.56,3.17)	0.009	27.9 %	0.239

For studies evaluating OS in different types of cancer, the results indicated that elevated miR-9 expression could estimate worse outcome in solid tumor and leukemia, with the pooled HR of 2.11(95 % CI: 1.44–3.10, *P* < 0.05), and 3.36(95 % CI: 1.22–9.29, *P* < 0.05) respectively (Fig. 3b). Significant heterogeneity existed across the studies within the subgroups.

The sample size did not alter the predictive value of miR-9 on the OS for all involved cancers. MiR-9 expression was found to be correlated with the outcome for sample sizes greater than or less than 100 subjects (HR = 2.02; 95 % CI: 1.44–2.82, *P* < 0.05 and HR = 2.70; 95 % CI: 1.13–6.44, *P* < 0.05, respectively) (Fig. 3c). There was significant heterogeneity across the studies within the subgroups.

Next, we examined the duration of follow-up and found that the follow-up months (more or less than 60 months) did not change the result of the estimated HR (HR = 2.32; 95 % CI: 1.52–3.53, *P* < 0.05 and HR = 1.68; 95 % CI: 1.14–2.48, *P* < 0.05, respectively) (Fig. 3d). There was significant heterogeneity across the studies within the subgroups.

Of note, miR-9 detection by qRT-PCR was demonstrated to be related with worse outcome in various neoplasms but not MISH method (HR = 2.54; 95 % CI: 1.72–3.76, *P* < 0.05 and HR = 1.07; 95 % CI: 0.45–2.57, *P* > 0.05, respectively) (Fig. 3e). Significant heterogeneity was detected within the subgroups.

Subsequently, we investigated the association between high level of miR-9 for the disease free survival (DFS), recurrence free survival and progress free survival (RFS and PFS) of patients with cancer. The random model was used because significant heterogeneity between these five multivariate analysis studies (I^2^ > 30.0 %). Elevated miR-9 expression exhibits no relevance to DFS, with the pooled HR of 2.54 (95 % CI: 0.92–7.06, *P* > 0.05). Increased high miR-9 levels was found moderately correlated with recurrence free survival/progress free survival (RFS/PFS) outcome in three multivariate analysis studies (HR: 2.08, 95 % CI: 1.33–3.27, *P* < 0.05) (Fig. 2c and d).

Overall, the combined HRs suggested that miR-9 expression may be an independent prognostic factor for patients with different kinds of cancer.

### miR-9 and Metastasis

**Fig. 4:**
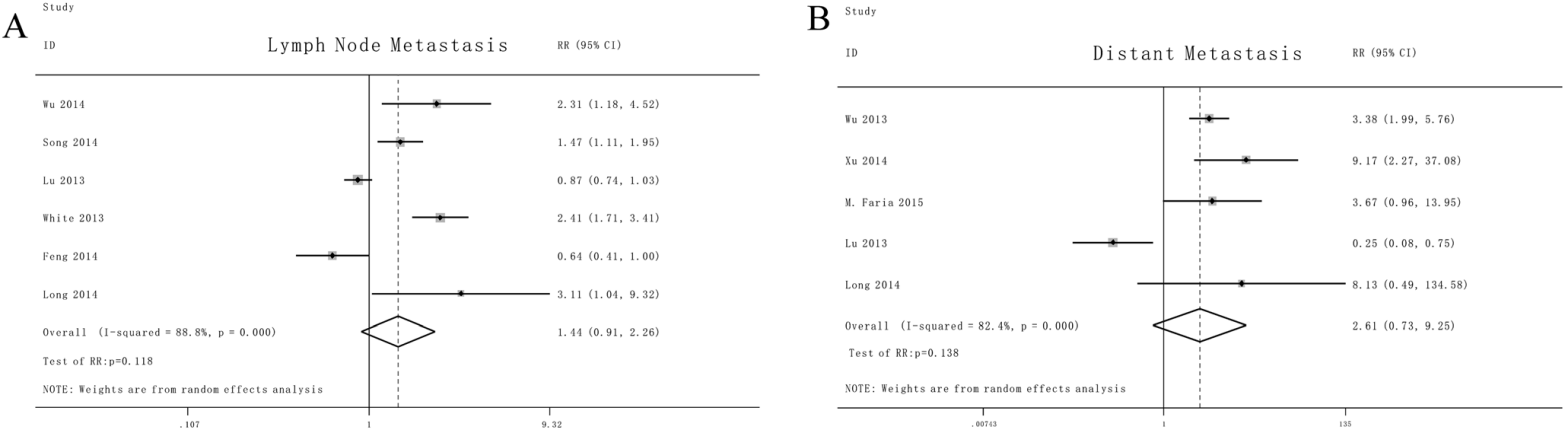
Forrest plots of lymph node metastasis (**a**) and distant metastasis (**b**)

We also investigated the association between miR-9 expression and cancer metastasis and the characteristics of the involved studies were summarized in Tables 3 and 4. When all the eligible articles were combined with random-effects models, as shown in Fig. 4, no significant associations were detected for lymph node metastasis (HR:1.44, 95 % CI: 0.91–2.26, *P* > 0.05) and distant metastasis (HR: 2.61, 95 % CI: 0.73–9.25, *P* > 0.05) respectively.

**Table 3:** Characteristics of studies included in the Lymph Node metastasis meta-analysis

Study	Year	No.of patients	Method	Cut-off	miR-9 High		miR-9 Low	
					Metastasis	Total	Metastasis	Total
Wu et al*.*	2014	103	qRT-PCR	Median	22	53	9	50
Song et al*.*	2014	243	MISH	>2 fold	45	82	60	161
Lu et al*.*	2013	150	qRT-PCR	Median	48	65	72	85
White et al*.*	2013	139	MISH	Median	50	63	25	76
Feng et al*.*	2014	50	qRT-PCR	1.0	9	16	30	34
Long et al*.*	2014	66	qRT-PCR	Score ≥ 4	20	45	3	21

**Table 4:** Characteristics of studies included in the distant metastasis meta-analysis

Study	Year	No.of patients	Method	Cut-off	miR-9 High		miR-9 Low	
					Metastasis	Total	Metastasis	Total
Wu et al*.*	2013	128	qRT-PCR	Median	46	68	12	60
Xu et al*.*	2014	79	qRT-PCR	Median	17	38	2	41
M. Faria et al*.*	2015	20	qRT-PCR	Median	6	9	2	11
Lu et al*.*	2013	150	qRT-PCR	Median	5	111	7	39
Long et al*.*	2014	66	qRT-PCR	Score ≥ 4	18	45	0	21

### Publication Bias

Publication bias of the included literatures was assessed by Begg’s plot and Egger’s tests. The tests revealed that no evidence of publication bias in the analysis of OS (Begg’s *P* = 1.000 and Egger’s *P* = 0.614) of all enrolled studies. The shapes of the funnel plots did not reveal any evidence of obvious asymmetry (Fig. S1).

### Sensitivity Analysis

Sensitivity analysis was conducted to investigate the effect of any single study on the overall outcomes. No significant difference was found after remove of any single study, suggesting that the conclusions are stable.

## Discussion

With the understanding and knowledge of miRs function in different kinds of tumor development and progression, scientists made tremendous contribution to the discovery that miRs could be a novel potential biomarker for cancer prognosis. According to the published data, miRs may potentially regulate up to 30 % of all protein coding genes and more than 50 % of total miRs are localized on the fragile region of the cancer genomics [29]. Furthermore, disparate miRs expression profiling is associated with various types of neoplasm and these miRs have been proved to play pivotal roles in tumorigenesis and progression. In addition, miRs is not vulnerable in easily obtained body fluid such as serum, plasma, and urine because miRs have extraordinary stability and tolerance even under severe physiochemical conditions including extreme temperature, PH, and freeze–thaw cycles [30, 31]. Collectively, the advantages mentioned above implied that the application of miRs as promising biomarkers for cancer prognosis is viable.

Whether miR-9 is oncogene or tumor suppressor had been discussed previously but the exact role of miR-9 in different cancers has not been fully elucidated. Recently, miR-9 has been discovered to be involved in modulating cellular processes through regulating the expression of several target genes such as E-cadherin [32], Foxo3a [33], NF-κB1 [34] and CXCR4 [35] et al. High expression levels of miR-9 were observed to promote multiple cancer cells proliferation, invasion and migration [36, 37], while knock-down of miR-9 could inhibit cancer cell growth in vitro [38]. In most clinical studies, as a widely accepted method in medical science, qRT-PCR was used to detect the miR-9 expression level. And elevated miR-9 was found in several types of cancers, including breast cancer, osteosarcoma, hepatocellular carcinoma, bladder cancer, glioma, laryngeal squamous cell carcinoma, non-small cell lung cancer, esophageal squamous cell carcinoma, et al.

After full texts of eligible literatures were reviewed, some studies found that a higher expression of miR-9 was significantly associated with worse outcome, such as overall survival, lymph node metastasis, distant metastasis, vascular involvement and high risk of recurrence. However, other studies presented insignificant or inverse results. For instance, Song et al*.* demonstrated that miR-9 promotes tumor metastasis and related to poor overall survival in EC, while the following study conducted by Feng et al*.* reached an opposite result that low expression of miR-9 related to lymph node metastasis of EC [16, 27]. These inconsistent results motivated us to perform a meta analysis to evaluate the relationship between miR-9 expression and the outcome of patients with various human cancers. Twenty publications comprising 2441 patients have been pooled in the present meta analysis, indicating a statistically significant role of miR-9 on OS (HR: 2.23, 95 % CI: 1.56–3.17, *P* < 0.05) and RFS/PFS (HR: 2.08, 95 % CI: 1.33–3.27, *P* < 0.05). Quite a few experts even recommended that the combination of miR-9 and other one or two miRs could better predict the prognosis of tumors. Mounting evidence from primary researches shed lights upon miR-9, through direct target gene E-cadherin, which may act as a pro-metastatic miRNA [32]. As shown in Fig. 4, no association was found between miR-9 expression and metastasis and the contradiction could be explained by the relative small numbers of eligible articles or the complicated mechanisms of different tumorigenesis and progression.

It should be prudentially make the conclusion of the association with miR-9 and tumors because the meta analysis has limitations and there are some issues should be considered. First, the cut-off value of miR-9 expression varied in different studies and it was arduous to reach a consensus value. Second, the analysis data derived from involved literatures should be translated with caution due to dramatic heterogeneity. As a matter of fact, the large heterogeneity of meta analysis may due to the differences in the clinical characteristics of patients (country, tumor stage etc.), the types of cancer, the types of specimen, the time of follow-up, and so on. Furthermore, the analysis result of our studies is not sufficiently persuasive, because the numbers of prognostic studies for survival and metastasis analyses were relatively small. We need more eligible clinical studies to confirm the relationship between miR-9 expression and prognosis of individuals with various cancers. The last but not the least, although no publication bias was detected in the meta analysis, potential publication bias may exist because desirable results might be published more easily, which can lead to an over estimation of overall outcome.

In our meta analysis study, even some limitations mentioned above, it was preliminarily concluded that promoted miR-9 level is effectively associated with the poor OS in various kinds of carcinoma. In the future, well designed clinical studies with unified cut-off value and larger samples should be carried out before the practical implementation of miR-9 on the prognosis of neoplasm patients.

## Electronic supplementary material

Below is the link to the electronic supplementary material.

Figure S1 Funnel plots of articles included in the meta-analyses of overall survival in multiple cancers. (GIF 17 kb)

High Resolution (TIF 68 kb)

Checklist S1 (DOC 56 kb)
