# Supplementary figures and images for: Prognostic Value of microRNA-9 in Various Cancers: a Meta-analysis
Source: Pathol Oncol Res. 2016 Nov 14;23(3):573–82. doi: 10.1007/s12253-016-0148-4 (PMC5487937; doi:10.1007/s12253-016-0148-4)

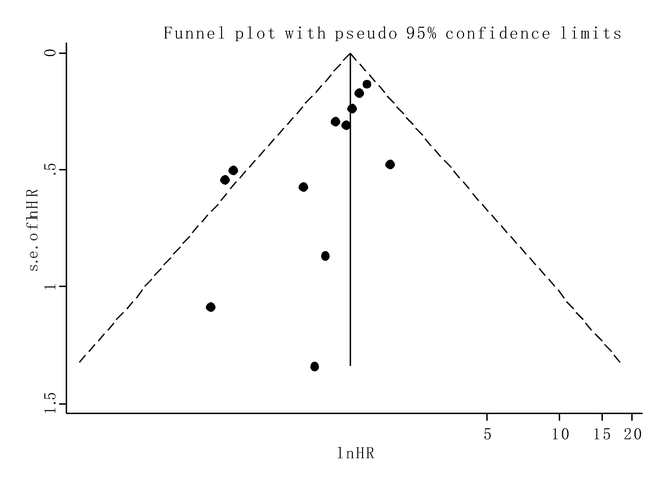

Supplement: Supplementary file 1 — Funnel plots of articles included in the meta-analyses of overall survival in multiple cancers. (GIF 17 kb) [file 12253_2016_148_Fig5_ESM.gif]
