# Supplementary material for: Prognostic Value of microRNA-9 in Various Cancers: a Meta-analysis
Source: Pathol Oncol Res. 2016 Nov 14;23(3):573–82. doi: 10.1007/s12253-016-0148-4 (PMC5487937; doi:10.1007/s12253-016-0148-4)
Supplement: Supplementary file 3 — (DOC 56 kb) [file 12253_2016_148_MOESM2_ESM.doc]

**Table S1**. MOOSE checklist

| **Reporting of background should include** | |
| --- | --- |
| Problem definition | Background |
| Hypothesis statement | Background |
| Description of study outcome(s) | OS, DFS, PFS, RFS, CSS, Lymph node metastasis, Distant metastasis |
| Type of exposure or intervention used | Various carcinomas |
| Type of study designs used | Meta analysis |
| Study population | Global |
| **Reporting of search strategy should include** | |
| Qualifications of searchers (eg, librarians and investigators) | Investigator |
| Search strategy, including time period included in the synthesis and keywords | Search strategy and Literature selection |
| Effort to include all available studies, including contact with authors | We contact authors and searched reference lists and citations. |
| Databases and registries searched | Material and Methods |
| Search software used, name and version, including special features used (eg, explosion) | IE 10 |
| Use of hand searching (eg, reference lists of obtained articles) | Search strategy and Literature selection |
| List of citations located and those excluded, including justification | Flow diagram in Figure 1. |
| Method of addressing articles published in languages other than English | Search strategy and Literature selection |
| Method of handling abstracts and unpublished studies | Material and Methods |
| Description of any contact with authors | Material and Methods |
| **Reporting of methods should include** | |
| Description of relevance or appropriateness of studies assembled for assessing the hypothesis to be tested | Material and Methods |
| Rationale for the selection and coding of data (eg, sound clinical principles or convenience) | Material and Methods |
| Documentation of how data were classified and coded (eg, multiple raters, blinding, and interrater reliability) | Material and Methods |
| Assessment of confounding (eg, comparability of cases and controls in studies where appropriate) | Material and Methods |
| Assessment of study quality, including blinding of quality assessors; stratification or regression on possible predictors of study results | Material and Methods |
| Assessment of heterogeneity | Material and Methods |
| Description of statistical methods (eg, complete description of fixed or random effects models, justification of whether the chosen models account for predictors of study results, dose-response models, or cumulative meta-analysis) in sufficient detail to be replicated | Material and Methods |
| Provision of appropriate tables and graphics | Methods and Results |
| **Reporting of results should include** | |
| Graphic summarizing individual study estimates and overall estimate | Figure 2, 3, 4 |
| Table giving descriptive information for each study included | Table 1. |
| Results of sensitivity testing (eg, subgroup analysis) | Sensitivity analysis |
| Indication of statistical uncertainty of findings | Discussion |
| **Reporting of discussion should include** | |
| Quantitative assessment of bias (eg, publication bias) | Publication bias |
| Justification for exclusion (eg, exclusion of non–English-language citations) | Inclusion and exclusion criteria |
| Assessment of quality of included studies | Quality Assessment |
| **Reporting of conclusions should include** | |
| Consideration of alternative explanations for observed results | Discussion |
| Generalisation of the conclusions (ie, appropriate for the data presented and within the domain of the literature review) | Discussion |
| Guidelines for future research | Discussion |
| Disclosure of funding source | Grant Support |
